# Linking erythropoietin to Treg-dependent allograft survival through myeloid cells

**DOI:** 10.1172/jci.insight.158856

**Published:** 2022-05-23

**Authors:** Julian K. Horwitz, Sofia Bin, Robert L. Fairchild, Karen S. Keslar, Zhengzi Yi, Weijia Zhang, Vasile I. Pavlov, Yansui Li, Joren C. Madsen, Paolo Cravedi, Peter S. Heeger

**Affiliations:** 1 Translational Transplant Research Center and Department of Medicine, Icahn School of Medicine at Mount Sinai, New York, New York, USA.; 2 Nephrology, Dialysis and Renal Transplant Unit, IRCCS - Azienda Ospedaliero-Universitaria di Bologna, Alma Mater Studiorum University of Bologna, Italy.; 3 Department of Immunology, the Cleveland Clinic, Cleveland, Ohio, USA.; 4 Center for Transplant Sciences and Division of Cardiac Surgery, Department of Surgery, Massachusetts General Hospital, Boston, Massachusetts, USA.

**Keywords:** Transplantation, Adaptive immunity, Cellular immune response, Mouse models

## Abstract

Erythropoietin (EPO) has multiple nonerythropoietic functions, including immune modulation, but EPO’s effects in transplantation remain incompletely understood. We tested the mechanisms linking EPO administration to prolongation of murine heterotopic heart transplantation using WT and conditional EPO receptor–knockout (EPOR-knockout) mice as recipients. In WT controls, peritransplant administration of EPO synergized with CTLA4-Ig to prolong allograft survival (*P <* 0.001), reduce frequencies of donor-reactive effector CD8^+^ T cells in the spleen (*P <* 0.001) and in the graft (*P <* 0.05), and increase frequencies and total numbers of donor-reactive Tregs (*P <* 0.01 for each) versus CTLA4-Ig alone. Studies performed in conditional EPOR-knockout recipients showed that each of these differences required EPOR expression in myeloid cells but not in T cells. Analysis of mRNA isolated from spleen monocytes showed that EPO/EPOR ligation upregulated macrophage-expressed, antiinflammatory, regulatory, and pro-efferocytosis genes and downregulated selected proinflammatory genes. Taken together, the data support the conclusion that EPO promotes Treg-dependent murine cardiac allograft survival by crucially altering the phenotype and function of macrophages. Coupled with our previous documentation that EPO promotes Treg expansion in humans, the data support the need for testing the addition of EPO to costimulatory blockade-containing immunosuppression regimens in an effort to prolong human transplant survival.

## Introduction

Despite improvements in the care of heart transplant recipients, outcomes remain suboptimal, with graft half-lives only approximating 11 years (1, 2). Cardiac allograft failure is commonly associated with T cell–dependent coronary vasculopathy with or without evidence of antibody-mediated injury, which implies inadequate immunosuppression and is a common contributor to graft loss. Calcineurin inhibitors (CNIs) comprise the core standard-of-care immunosuppression for heart transplantation in the United States and are highly effective at inhibiting T cells. However, their off-target morbidities, including kidney toxicity, are significant, highlighting the need for developing novel treatment strategies to improve heart transplant outcomes (1, 3). Importantly, CNIs have been shown to nonspecifically inhibit all T cell subsets, including Tregs (4, 5).

CD4^+^ Tregs are vital negative regulators of alloresponses induced to transplanted organs. They suppress the functions of pathogenic alloreactive T cells, are essential for transplant tolerance in preclinical models, and are associated with improved transplant outcomes in human transplant recipients (6–11). Studies initially performed in mouse models showed that CNI administration prevents Treg-dependent allograft tolerance, and translational work in humans demonstrated dysfunctional Tregs in CNI-treated transplant recipients (4, 5, 12). Identification of new CNI-free treatment strategies that facilitate Treg function is thus an important goal for the field.

While promising (13, 14), the efficacy of adoptively transferring ex vivo expanded Tregs into organ transplant recipients is unproven. Furthermore, the approach is costly and labor intensive, lowering the likelihood that it will become routinely available in the near future. Development of distinct in vivo pharmacological approaches that inhibit the function of pathogenic effector T cells while promoting graft-protective properties of Tregs in the absence of CNIs would address this unmet medical need and if achieved, could be transformative.

Erythropoietin (EPO) is a hormone produced predominantly by kidney perivascular interstitial fibroblasts but also by fetal liver cells and monocytes/macrophages (15). It stimulates hematopoiesis via ligation of a homodimeric EPO receptor (EPOR) on RBC precursors that initiates a JAK2/STAT5-dependent signaling cascade and prevents their apoptosis (15). Emerging evidence from our research group, among others, showed EPO/EPOR ligation has pleiotropic immunoregulatory properties that are distinct from EPO’s ability to stimulate RBC production (16–19). These studies showed that EPO binds to the homodimeric EPOR expressed on T cells and antigen-presenting cells (APCs) to (a) inhibit proliferation of naive and memory CD4^+^ and CD8^+^ T cells; (b) prevent CD4^+^ Th1, Th17, and Tfh cell differentiation and expansion; and yet (c) facilitate Treg generation and expansion.

The protolerogenic mechanisms initiated by EPOR signaling suggest that promoting EPOR activation on immune cells could be a Treg-promoting immunosuppression strategy capable of improving heart transplant survival and potentially inducing transplant tolerance. Herein, we employed a preclinical murine system to test this hypothesis.

## Results

**Figure 1:**
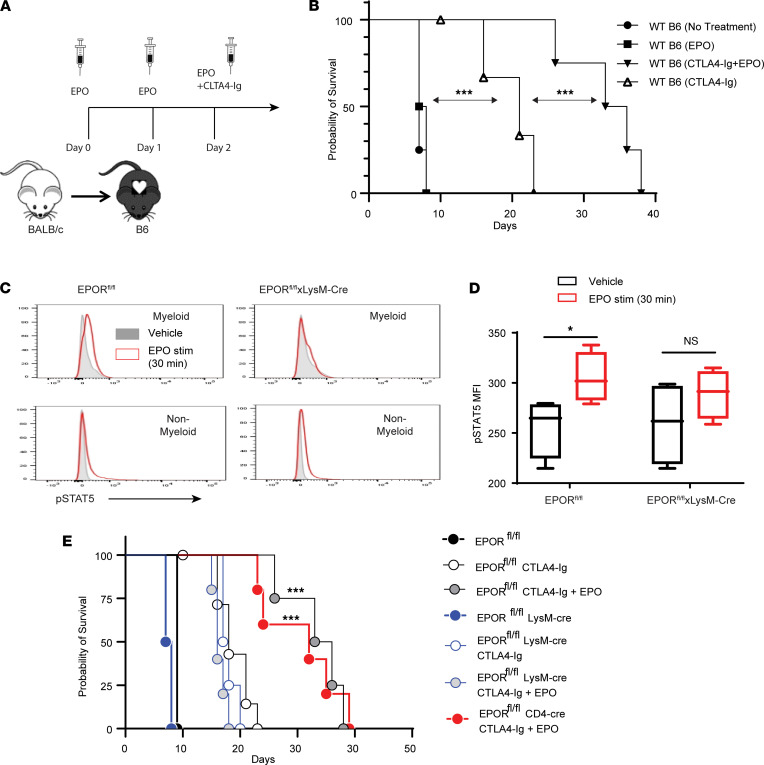
EPO synergizes with CTLA4-Ig to prolong heart transplant survival through ligation of EPOR expressed on myeloid cells. (**A**) Schematic of design and treatment regimen. (**B**) Survival of BALB/c hearts transplanted into WT B6 recipients and either not treated (*n =* 3) or treated with EPO alone (*n =* 3), CTLA4-Ig alone (*n =* 4), or EPO+CTLA4-Ig (*n =* 5). Results compared among groups by Kaplan-Meier (log-rank) survival analysis. (**C**) Representative flow cytometry plots of intracellular pSTAT5 in splenic myeloid or nonmyeloid cells obtained from EPOR^fl/fl^ or EPOR^fl/fl^ LysM-Cre animals 30 minutes after in vitro treatment with vehicle or EPO as indicated. (**D**) Quantified results (*n =* 3/group) of pSTAT5 in splenic myeloid cells isolated from EPOR^fl/fl^ or EPOR^fl/fl^ LysM-Cre animals 30 minutes after in vitro treatment with vehicle or EPO. Statistics in **D** compared by 2-tailed Student’s *t* test. Representative of 2 independent experiments. (**E**) Survival of BALB/c hearts transplanted into B6 EPOR^fl/fl^, EPOR^fl/fl^ LysM-Cre, or EPOR^fl/fl^ CD4-Cre recipients and treated with or without CTLA4-Ig and with or without EPO as indicated. Results compared among groups by Kaplan-Meier (log-rank) survival analysis. *n =* 6 per group except for untreated EPOR^fl/fl^ and EPOR^fl/fl^ LysM-Cre (*n =* 3/group). *P < 0.05; ***P < 0.001; NS, not significant.

Building upon our previous findings (19), we tested the effects of a single dose of CTLA4-Ig plus short-term recombinant EPO therapy as a CNI-free strategy to prolong fully MHC-disparate cardiac allograft survival. We transplanted BALB/c hearts into B6 recipients with or without EPO (5000 U/day for 3 days) and with or without 150 μg of CTLA4-Ig on day 2 after transplant (Figure 1A). Graft survival analyses (Figure 1B) showed that CTLA4-Ig alone increased median survival time (MST) to 17 days versus 7 days in untreated (or IgG control treated) recipients (*P <* 0.05). Peritransplant EPO therapy alone (without CTLA4-Ig) had no effect (MST 8 days, *P =* NS vs. untreated controls, *P <* 0.05 vs. CTLA4-Ig), but the same 3-day peritransplant EPO administration synergized with the CTLA4-Ig to prolong graft survival to 34 days (*P <* 0.05 vs. CTLA4-Ig alone).

To discern mechanisms underlying this effect, we crossed our previously generated B6 EPOR conditional knockout mouse (EPOR^fl/fl^; ref. 18) with mice that express Cre under control of the LysM promoter (EPOR^fl/fl^ LysM-Cre), generating animals that lacked EPOR on myeloid cells. Studies of EPOR signaling on myeloid cells isolated from these animals confirmed specific absence of EPOR-induced STAT5 phosphorylation (Figure 1, C and D). Transplantation of BALB/c hearts into groups of CTLA4-Ig/EPO–treated EPOR^fl/fl^ recipients and similarly treated and previously verified EPOR^fl/fl^ CD4-Cre (18) recipients (lack EPOR on essentially all CD4^+^ and CD8^+^ T cells; ref. 20) resulted in equivalent prolonged heart graft survival (MST 32 days, *P =* NS, Figure 1E), indicating that T cell–expressed EPOR was not required for the EPO-induced graft prolongation. In contrast, when we transplanted BALB/c hearts into CTLA4-Ig/EPO–treated EPOR^fl/fl^ LysM-Cre recipients (Figure 1E), we observed shortened graft survival versus either of the other groups. EPO no longer synergized with CTLA4-Ig, indicating that EPOR on myeloid cells was required for the synergistic enhancement of allograft survival by EPO plus CTLA4-Ig treatment (MST 18 days, *P =* NS vs. CTLA4-Ig–treated EPOR^fl/fl^ controls). Control studies showed untreated EPOR^fl/fl^ and EPOR^fl/fl^ LysM-Cre recipients rejected BALB/c hearts by day 9, not different from the WT B6 controls shown in Figure 1B.

**Figure 2:**
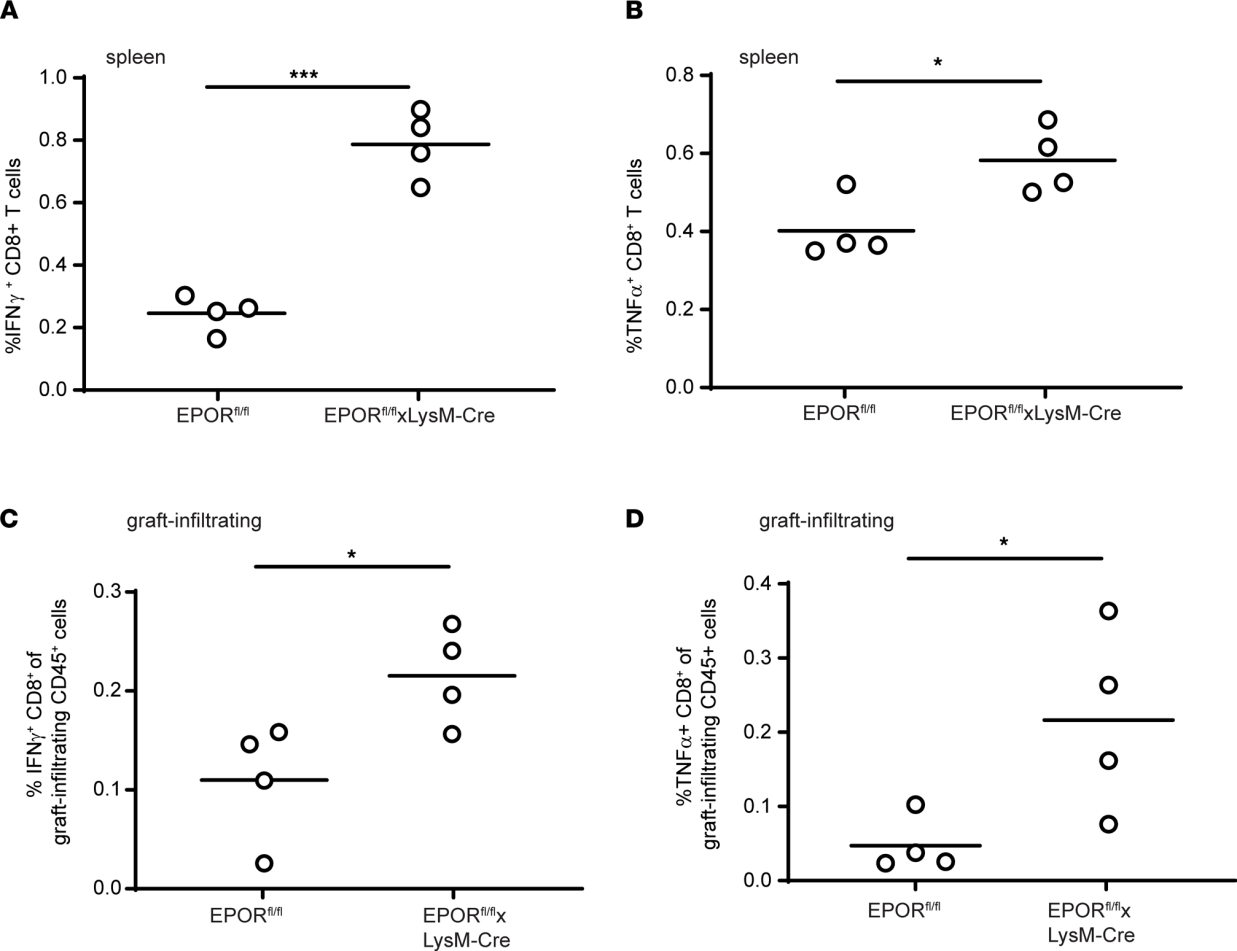
Absence of EPOR from recipient myeloid cells augments donor-reactive T cell immunity. (**A** and **B**) Frequencies of splenic donor-reactive IFN-γ (**A**) and TNF-α producers (**B**) analyzed by flow cytometry on day 14 after transplanting BALB/c hearts into B6 EPOR^fl/fl^ or EPOR^fl/fl^ LysM-Cre recipients treated with a single dose of CTLA4-Ig plus EPO as in Figure 1A. (**C** and **D**) Frequencies of graft-infiltrating IFN-γ– (**C**) and TNF-α– producing (**D**) lymphocytes (flow cytometry) on day 14 after transplanting BALB/c hearts into B6 EPOR^fl/fl^ or EPOR^fl/fl^ LysM-Cre recipients treated with a single dose of CTLA4-Ig plus EPO as in Figure 1A. Each symbol is mean of 2 to 3 replicates. For each panel, results between the 2 groups were analyzed using 2-tailed Student’s *t* test. *P < 0.05; ***P < 0.001.

**Figure 3:**
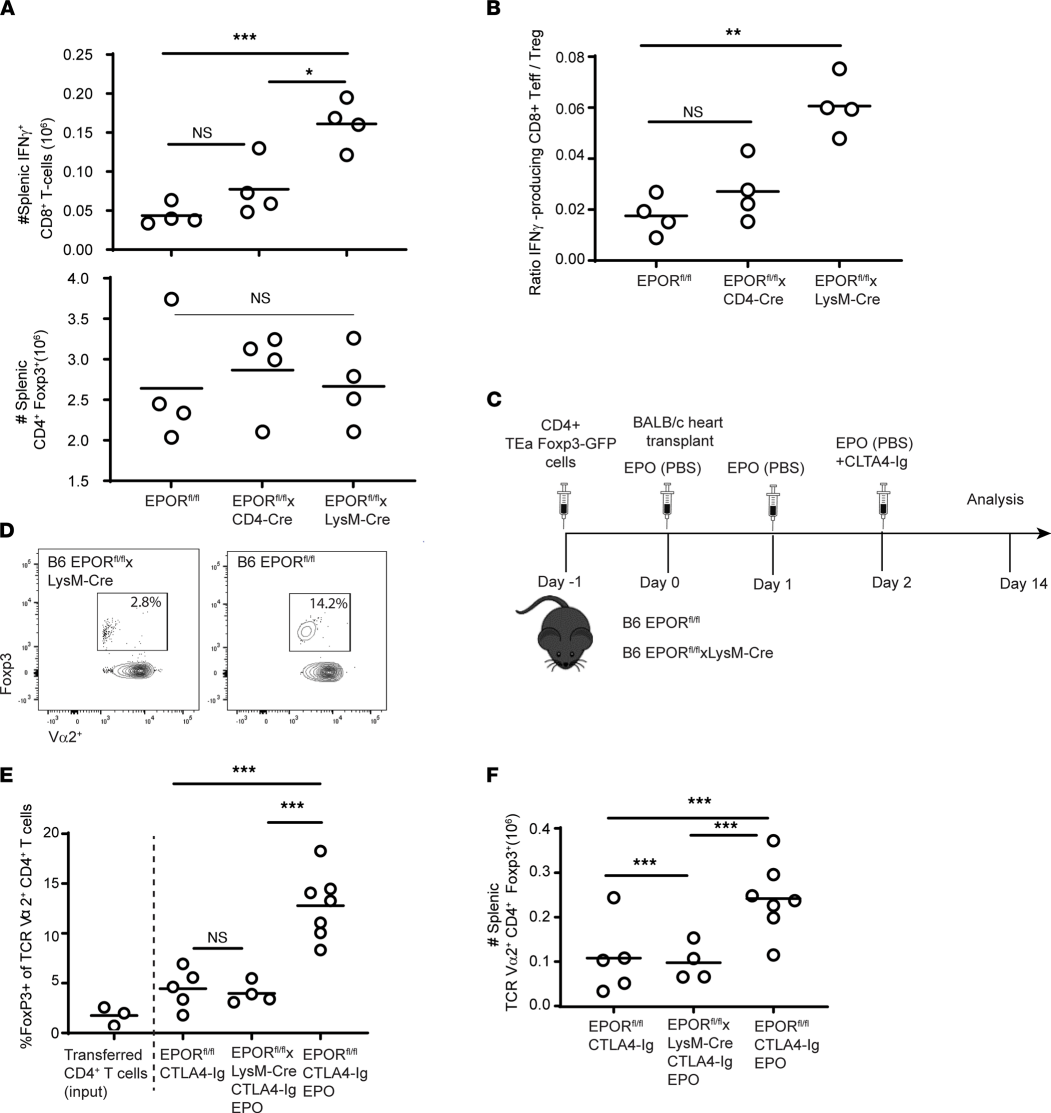
EPO ligation of myeloid cell EPOR expression expands peripheral donor-reactive CD4^+^Foxp3^+^ T cells. (**A** and **B**) Numbers of splenic donor-reactive IFN-γ–producing CD8^+^ T cells (top) and CD4^+^Foxp3^+^ T cells (bottom) by flow cytometry, and calculated ratios of CD8^+^ IFN-γ–producing CD8^+^ and CD4^+^Foxp3^+^ T cells (Tregs) in each animal (**B**) on day 14 after transplanting BALB/c hearts into B6 EPOR^fl/fl^, EPOR^fl/fl^ LysM-Cre, and EPOR^fl/fl^ CD4-Cre recipients (all treated with a single dose of CTLA4-Ig plus EPO as in Figure 1A). (**C**) Schematic of experimental design. (**D**–**F**) Representative flow plots showing Foxp3 expression gated on CD4^+^Vα2^+^ cells (**D**), quantified frequencies of splenic CD4^+^Foxp3^+^Vα2^+^ TEa T cells (**E**), and absolute numbers of splenic CD4^+^Foxp3^+^Vα2^+^ TEa T cells (**F**) on day 14 after adoptive transfer into EPOR^fl/fl^ or EPOR^fl/fl^ LysM-Cre recipients of BALB/c heart grafts treated with CTLA4-Ig with or without EPO as indicated. Each symbol represents the mean of *n =* 2 to 3 replicate assays from each animal. Comparisons performed using 1-way ANOVA corrected for multiple comparisons among paired groups. *P < 0.05; **P < 0.01; ***P < 0.001; NS, not significant. Teff, effector T cell.

When we analyzed frequencies of donor-reactive IFN-γ– and TNF-α–producing T cells from spleens of additional groups of EPOR^fl/fl^ and EPOR^fl/fl^ LysM-Cre recipients on day 14 after transplant, we observed higher frequencies in the CTLA4-Ig/EPO–treated EPOR^fl/fl^ LysM-Cre recipients (Figure 2, A and B). Analyses of graft-infiltrating, donor-reactive, IFN-γ– and TNF-α–producing CD8^+^ T cells also revealed higher frequencies in the EPO/CTLA4-Ig–treated EPOR^fl/fl^ LysM-Cre mice versus the EPO/CTLA4-Ig–treated EPOR^fl/fl^ controls (Figure 2, C and D). To assess effects of EPO on expansion of donor-reactive CD8^+^ T cells and CD4^+^Foxp3^+^ Tregs, we next quantified total numbers of each of these populations in the spleens of EPOR^fl/fl^, EPOR^fl/fl^ CD4-Cre, and EPOR^fl/fl^ LysM-Cre heart allograft recipients treated with CTLA4-Ig/EPO on day 14 after transplant (Figure 3, A and B). These analyses showed higher absolute numbers of donor-reactive IFN-γ producers in the EPOR^fl/fl^ LysM-Cre recipients, yet no differences between those from EPOR^fl/fl^ and EPOR^fl/fl^ CD4-Cre recipients. The analyses also showed no differences in total numbers of CD4^+^Foxp3^+^ T cells among the groups. Calculated ratios of donor-reactive CD8^+^ T cells to CD4^+^Foxp3^+^ T cells showed significantly higher values in the spleens of EPO-treated EPOR^fl/fl^ LysM-Cre versus EPOR^fl/fl^ or EPOR^fl/fl^ CD4-Cre recipients (Figure 3B). Together, the data indicated that EPO reduced donor-reactive T cell expansion and did so via ligating EPORs expressed on myeloid cells, not through directly stimulating T cell–expressed EPOR.

Although we did not observe differences among the groups of transplant recipients in the frequencies or total numbers of the splenic CD4^+^Foxp3^+^ T cell repertoire, it remained possible that after transplantation, EPO/EPOR ligation on myeloid cells promoted differentiation/expansion of the subset of donor-reactive Tregs (which then contributed to the reduced expansion of effector T cells). To directly test this hypothesis in vivo, we adoptively transferred purified Foxp3-GFP^+^TEa^+^CD4^+^ T cell receptor–transgenic (TCR-transgenic) T cells (*H-2^b^*, Vα2^+^ TCR reactive to I-A^b^ plus a peptide from the donor I-E^d^α chain) into groups of EPOR^fl/fl^ and EPOR^fl/fl^ LysM-Cre mice (Figure 3C, schematic) followed by transplantation with BALB/c hearts and treatment with CTLA4-Ig/EPO as in the previous experiments. When we quantified Foxp3-GFP positivity within the TEa cells on day 14 after transplant (Figure 3, D and E), we observed that treatment with CTLA4-Ig alone induced an approximately 50% increase in the frequency of Foxp3^+^Va2^+^CD4^+^ T cells in the EPOR^fl/fl^ allograft recipients (noting that the adoptively transferred TEa cells contained 3% Foxp3-GFP^+^ cells at baseline). CTLA4-Ig plus EPO induced a 3- to 4-fold increase in the frequencies of Foxp3^+^Vα2^+^CD4^+^ T cells in EPOR^fl/fl^ recipients. In contrast, frequencies of Foxp3^+^Vα2^+^CD4^+^ T cells in CTLA4-Ig/EPO–treated EPOR^fl/fl^ LysM-Cre recipients did not differ from control animals treated with CTLA4-Ig alone. Quantification of the absolute numbers of splenic Foxp3^+^Va2^+^CD4^+^ T cells in each animal (Figure 3F) showed significantly more donor-reactive Foxp3^+^ TEa cells in the CTLA4-Ig/EPO–treated EPOR^fl/fl^ recipients versus either CTLA4-Ig/EPO–treated EPOR^fl/fl^ LysM-Cre recipients or the EPOR^fl/fl^ recipients treated with CTLA4-Ig (no EPO).

**Figure 4:**
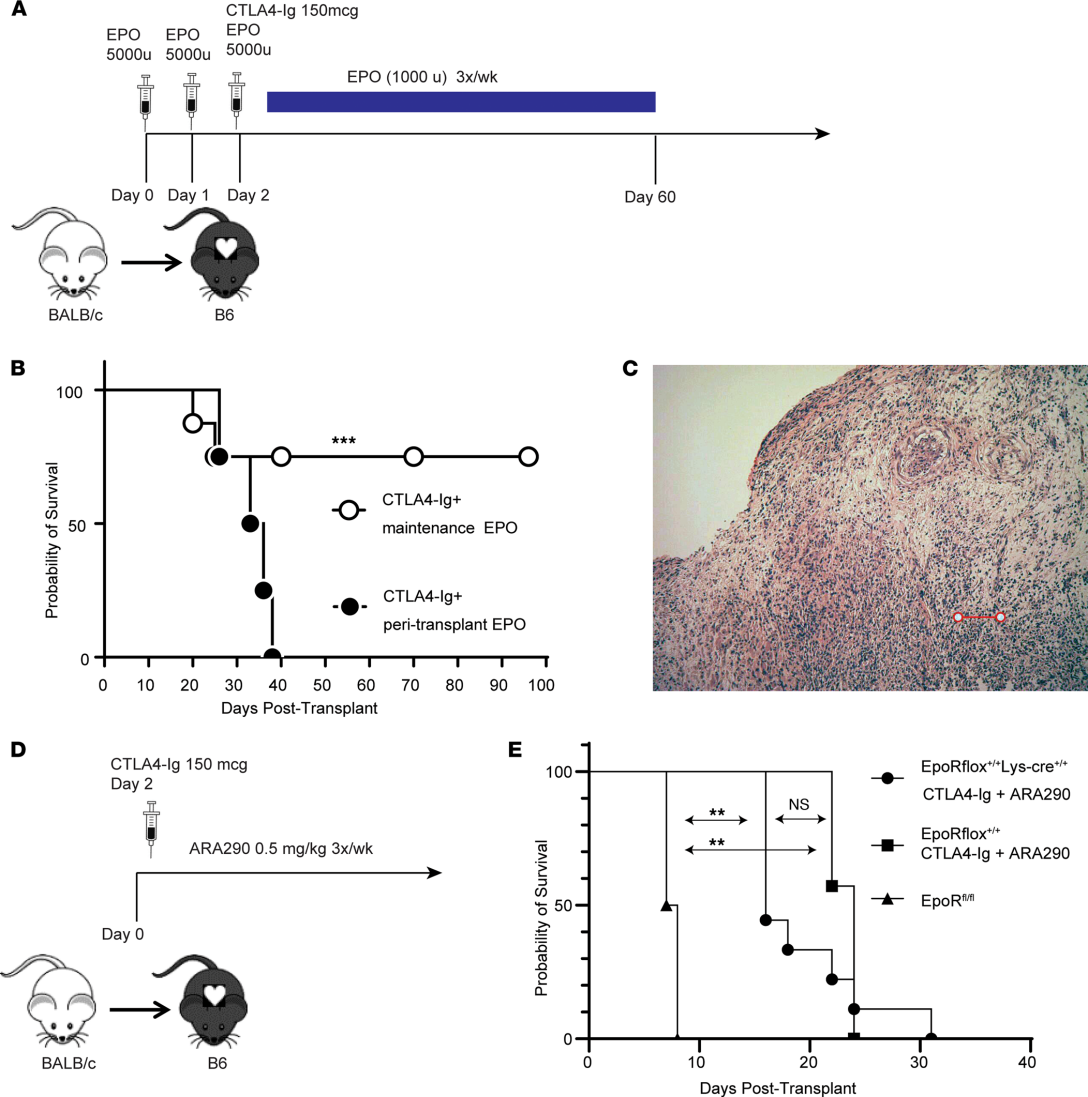
Chronic treatment with EPO but not with a nonerythropoietic EPOR agonist ARA290 prolongs heart graft survival. (**A**) Schematic of treatment protocol for maintenance EPO administration. (**B**) Kaplan-Meier survival analysis of BALB/c hearts transplanted into CTLA4-Ig–treated recipients plus EPO either peritransplant or 3 times per week until day 60 (maintenance). (**C**) Representative photomicrograph of H&E-stained allograft from a recipient given chronic maintenance EPO through day 60 and harvested on day 90 after transplant, showing diffuse mononuclear cell infiltration consistent with rejection (scale bar: 200 μm). (**D**) Schematic of treatment protocol for ARA290 administration. (**E**) Kaplan-Meier survival analysis of BALB/c hearts transplanted into CTLA4-Ig–treated recipients plus ARA290 3 times per week. **P < 0.01; ***P < 0.001; NS, not significant.

Understanding that induction therapy with EPO prolongs myeloid cell–dependent graft survival and expanded populations of donor-reactive Tregs, we next tested whether prolonged EPO administration induced immune tolerance. We transplanted BALB/c hearts into CTLA4-Ig–treated recipients and treated the animals with EPO 3 times per week for 60 days, monitoring hematocrits and holding EPO doses when hematocrits increased to more than 60% (Figure 4A). These experiments showed heart grafts survived beyond 100 days in 8 of 10 recipients (Figure 4B). Nonetheless, histological examination of beating allografts on day 65 showed diffuse mononuclear cell infiltration (3 of 4 analyzed) and vasculopathy (2 of 4 analyzed), consistent with acute and chronic allograft rejection (Figure 4C).

In addition to signaling through EPOR homodimers, EPO binds to a low affinity heterodimer composed of 1 EPOR chain and the β common receptor chain CD131 (15, 21). Evidence suggests that EPO binding to EPOR/CD131 heterodimers can mediate some of EPO’s nonerythropoietic effects, including those involved in tissue and immune homeostasis and repair (15, 21–23). ARA290 is a nonerythropoietic EPO derivative that specifically ligates the EPOR/CD131 heterodimer with high affinity; it does not bind to or signal via the EPOR homodimer (24, 25). We administered ARA290 at 0.5 mg/kg 3 times a week to CTLA4-Ig–treated EPOR^fl/fl^ and EPOR^fl/fl^ LysM-Cre recipients of BALB/c heart grafts, a dose previously shown to be effective in vivo in other murine models (refs. 24, 26–28; Figure 4D). These experiments showed that the addition of ARA290 did not prolong heart graft survival beyond that induced by CTLA4-Ig regardless of EPOR expression on myeloid cells (Figure 4E). This result supports the conclusion that EPO’s therapeutic effects in this transplant system require EPO/EPOR homodimer ligations and do not involve the CD131/EPOR heterodimer.

**Figure 5:**
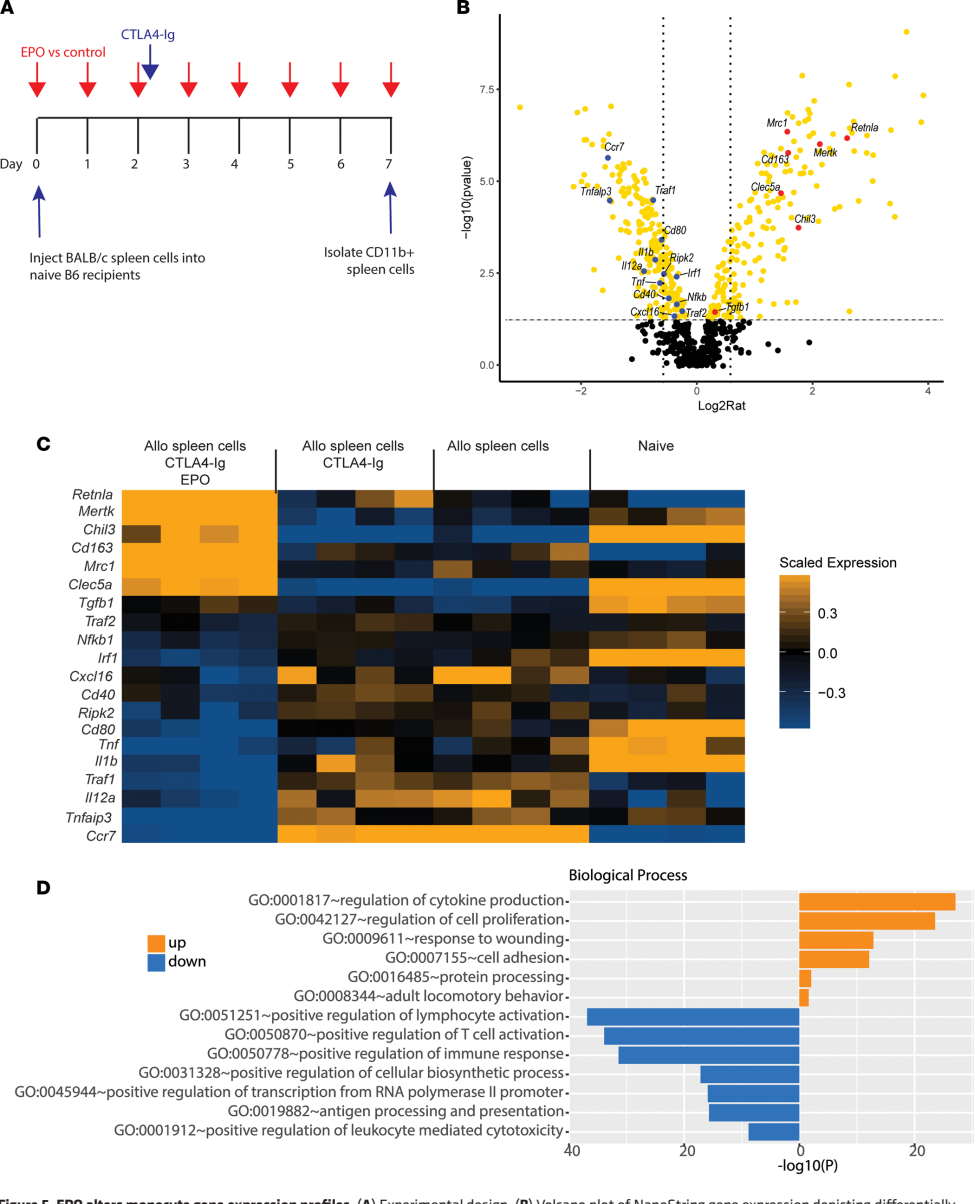
EPO alters monocyte gene expression profiles. (**A**) Experimental design. (**B**) Volcano plot of NanoString gene expression depicting differentially expressed genes (yellow) by Limma test (upregulated to the right) for monocytes isolated from B6 mice injected with BALB/c spleen cells/CTLA4-Ig/EPO versus BALB/c spleen cells/CTLA4-Ig, *n* = 4/group. Selected upregulated or downregulated genes are annotated in red or blue, respectively; *x* axis fold-change, vertical lines drawn at 1.5-fold; *y* axis adjusted *P* value, horizontal line drawn at adjusted *P* value = 0.05. (**C**) Multigroup heatmap demonstration of key upregulated or downregulated genes. (**D**) Bar chart of significantly enriched biological processes of differentially upregulated or downregulated genes comparing BALB/c spleen cells/CTLA4-Ig/EPO with BALB/c spleen cells/CTLA4-Ig.

To provide additional insight into mechanisms linking myeloid cell EPOR signaling to the observed prolongation of allograft survival, we profiled splenic monocyte gene expression in naive B6 mice with or without CTLA4-Ig and with or without EPO after i.v. administration of BALB/c spleen cells (Figure 5A schematic, *n =* 4/group), a stimulus we previously showed induces potent pathogenic alloimmune responses (29). Seven days later, we isolated RNA from splenic CD11b^+^CD11c^–^ myeloid cells and quantified and compared gene expression profiles among the groups using a macrophage-gene–enriched NanoString code set. Analysis of differentially expressed genes among groups showed more than 500 differentially expressed genes (by 1-way ANOVA, Supplemental Figure 1 and Supplemental Table 1). Comparison of gene expression between recipients of BALB/c spleen cells/CTLA4-Ig versus BALB/c spleen cells/CTLA4-Ig/EPO (Figure 5B) showed EPO downregulated 190 genes and upregulated 228 genes.

Among the upregulated genes are those associated with various immunoregulatory/antiinflammatory macrophage phenotypes: *Retnla* and *Chil3* (also known as *Ym1*) are signature M2 genes (30–33), *Mertk* and *Mrc1* are involved in efferocytosis (34), and *CD163* and *Clec5* are expressed in alternatively activated macrophages or regulatory macrophages (35, 36). Of the genes significantly downregulated by EPO are those associated with proinflammatory/M1-like macrophages (30), including *Ccr7*, *Tnfaip3*, *Traf1*, *Cd80*, and *IL-12a* (Figure 5B). Comparative analysis of genes among treatment groups (Figure 5C) showed that for approximately 20 of the depicted genes, injection of allogeneic spleen cells altered their expression versus naive controls; additional treatment with CTLA4-Ig did not have an effect, while addition of EPO markedly altered their expression. Gene ontology analyses of all differentially expressed genes between animals treated with allogeneic spleen cells and CTLA4-Ig with or without EPO (Figure 5D), revealed EPO administration upregulated genes within the biological processes of response to wounding, regulation of cytokine production, and regulation of cell proliferation, among others. The biological processes downregulated by EPO (comparing BALB/c spleen cells/CTLA4-Ig vs. BALB/c spleen cells/CTLA4-Ig/EPO) included positive regulation of lymphocyte activation, positive regulation of T cell activation, positive regulation of immune response, positive regulation of leukocyte-mediated cytotoxicity, and positive regulation of lymphocyte activation, all consistent with an antiinflammatory macrophage profile.

## Discussion

Increasing evidence indicates that EPO has pleiotropic effects on various immune cells (15, 17–19, 37–39), yet the mechanisms underlying EPO-induced allograft survival have remained unclear. Although previously published in vitro studies showed that EPO can directly inhibit effector T cell expansion, differentiation, and function by ligating T cell–expressed EPOR (16, 19), our newly generated data in murine recipients of cardiac allografts did not support this mechanism; recipient absence of T cell–expressed EPOR did not alter kinetics of heart graft survival, nor did it alter the strength/cytokine profiles of the induced donor-reactive effector T cell responses when compared with control WT recipients (that expressed EPOR on T cells). Rather, our current studies using conditional knockout mice as heart allograft recipients showed that EPO/EPOR ligations specifically on myeloid cells (a) reduced frequencies of donor-reactive effector T cells, (b) augmented frequencies and absolute numbers of donor-reactive Tregs, (c) induced regulatory/antiinflammatory cell gene expression profiles in macrophages, and (d) conferred long-term cardiac allograft survival. The data support the conclusion that the effects of EPO on donor-reactive T cell immunity in this transplant system were indirect; induction of a set of protective macrophages promoted donor-reactive Treg expansion that together limited generation/function of a pathogenic effector T cell response to promote graft survival.

Our data also provide insight into the complexities of the EPO-induced effects on macrophages. The gene expression profiling analyses indicated that the EPO-induced, protective macrophage phenotype did not fit the traditional paradigm of proinflammatory M1 versus antiinflammatory (pro-healing) M2 macrophage subsets originally described in the early 2000s (40). We found that EPO administration did reduce expression of a subset of genes known to be involved in recruitment and differentiation of proinflammatory M1-like macrophages (*Ccr7*, *Tnfaip3*, *Traf1*, *Cd80*, and *IL-12a*) and did upregulate a subset of signature antiinflammatory M2 macrophage genes, including *Retna* and *Chil3* (also called *Ym1*). We additionally observed that EPO upregulated macrophage production of efferocytosis-related molecules *Mertk* and *Mrc1* in response to allogeneic stimuli, suggesting a role for macrophage-mediated efferocytosis, i.e. quiescent clearance of apoptotic/dead cells (41), in prolonging transplant survival. The data also indicated that EPO induced some genetic and functional signatures characteristic of regulatory macrophages, which we and others have shown can inhibit effector T cells as well as promote generation and expansion of suppressive donor-reactive Tregs (19, 42).

We speculate that these EPO-induced, heterogeneous gene expression alterations likely reflect multiple macrophage subsets with distinct functional and migratory abilities, which together are required for the protective effects in this transplant system. Although this hypothesis will require additional study, we note that previous work by others uncovered varied yet distinct effects of EPO on macrophage function in various other disease models. EPO was shown to promote antiinflammatory and M2 gene expression profiles in murine models of acute kidney injury (43) and experimental colitis (44, 45) and was linked to macrophage efferocytosis in a model of kidney nephritis (37). EPO has also been shown to increase frequencies of Tregs in murine experimental allergic encephalomyelitis (42), although a role for macrophages was not addressed. Thus, our data implicating broad and varied effects of EPO on macrophage gene expression and implied function provide insight into the aforementioned publications by showing that EPO has the ability to reduce inflammation and improve outcomes through all of the mechanisms observed in these various disease model systems.

Independent of a role for EPO, our research group among others previously provided mechanistic links among macrophage subset differentiation and graft outcomes. Costimulatory blockade promotes induction of regulatory macrophages capable of inhibiting effector T cells and fostering expansion of Tregs (36, 46). Additional work showed that exposure to various in vivo stimuli causes epigenetic alterations in macrophages that result in a pathogenic, “trained,” innate memory macrophage phenotype (47) that prevents allograft tolerance. Moreover, therapeutic strategies targeting activation of trained immunity can promote generation of regulatory macrophages (47), which are essential for inducing transplant tolerance in mice (36). Our data add to these findings by demonstrating that EPO/EPOR signaling on macrophages is one signal capable of shifting the balance toward immune regulation.

We note that while the combination of EPO and CTLA4-Ig tested herein had potent and broad immunoregulatory effects on myeloid cells that resulted in prolonged graft survival during its administration, the immunomodulation protocol that we used did not reproducibly result in immune tolerance. Nonetheless, our findings demonstrated proof of principle that this treatment strategy, if optimized, could be applied clinically for prevention of transplant rejection and could potentially be effective as a therapy for selected T cell–dependent autoimmune diseases. Because EPO promotes expansion of donor-reactive Tregs in vivo, EPO administration is a therapeutic strategy that could bypass the complexities and expense required for ex vivo Treg expansion and reinfusion in these contexts. In fact, pilot studies performed in patients with chronic kidney disease (19) and patients with autoimmune liver disease (48) indicate that clinically employed doses of EPO reliably increase frequencies of peripheral blood Tregs.

In sum, our studies provide evidence that EPO promotes graft survival in mice through inducing a protective myeloid cell program that promotes Treg expansion and limits donor-reactive effector T cell differentiation and function. Together with our previous work, the data support the need for testing the addition of EPO to costimulatory blockade–containing immunosuppression to prevent rejection and prolong graft survival in human transplant recipients.

## Methods

### Animals.

We purchased C57BL/6J (B6, *H-2^b^*) (catalog 000664) and BALB/cJ (*H-2^d^*) (catalog 000651) from The Jackson Laboratory or bred them from Jackson Laboratory–derived animals at the Icahn School of Medicine at Mount Sinai. Generation of EPOR^fl/fl^ mice was described previously (18). We crossed them with B6 mice expressing Cre under the CD4 promoter or under the LysM (catalog 018956) promoter (both from The Jackson Laboratory) to produce mice lacking EPOR on T cells or myeloid cells, respectively. CD4-Cre (catalog 022071) is expressed in CD4^+^CD8^+^ (double positive) thymocytes (20), which are precursors to both mature CD4^+^ and mature CD8^+^ peripheral blood T cells. We obtained TCR-Tg TEa mice (CD4^+^ reactive to I-A^b^ *+* I-Eα_52–68_) (49, 50) as a gift from Alexander Rudensky (Memorial Sloan Kettering Cancer Center) and bred them at Mount Sinai. We housed all animals in the Center for Comparative Medicine and Surgery at the Icahn School of Medicine at Mount Sinai under Institutional Animal Care in accordance with guidelines of the Association for Assessment and Accreditation of Laboratory Animal Care International. Experiments were performed with age- and sex-matched mice and using animals that were littermates or were maintained in the same room and/or were cohoused within the same cages for more than 2 weeks to limit potential effects of microbiome differences.

### Murine heterotopic heart transplantation.

The microsurgery core in the Icahn School of Medicine at Mount Sinai performed the heterotopic heart transplantation procedures as previously described (51, 52). We defined graft failure as the day on which a palpable heartbeat was no longer detectable, and we confirmed that the graft loss was due to rejection by histological examination of the graft tissue. In selected experiments, recipient mice received 150 μg CTLA4-Ig (Abatacept, Bristol-Myers Squibb) i.p. once on postoperative day 2. For experiments testing the effects of short-term EPO administration, recipient mice received 5000 U/kg of recombinant EPO (epoetin alfa, Amgen) i.p. daily on postoperative day 0 (pretransplant) through postoperative day 2. The effects of maintenance EPO administration were evaluated by administering 5000 U/kg of epoetin alfa i.p. daily on postoperative day 0 (pretransplant) through postoperative day 2 followed by 1000 U/kg of epoetin alfa i.p. 3 times per week. In selected studies, we administered CTLA4-Ig on day 2 plus the nonerythropoietic EPOR agonist ARA290 (Araim Pharmaceuticals) at a dose of 0.5 mg/kg i.p. 3 times per week.

### Cell isolations.

Mouse spleens were passed through a 40 μm strainer (BD Falcon, Corning) to make a single-cell suspension and subsequently lysed with RBC lysis buffer (Life Technologies). Splenic APC enrichment (kit 18951, CD90.2^+^ T cell bead depletion), splenic monocyte isolation (kit 19861, negative selection), splenic CD4^+^ T cell isolation (kit 19852, negative selection), and splenic CD11b^+^ myeloid cell isolation (kit 18970, positive selection) were performed by magnetic bead isolation as per the manufacturer’s instructions (STEMCELL). For heart graft–infiltrating lymphocyte analysis, grafts were minced with scissors and treated with collagenase A (Sigma-Aldrich, 1 mg/mL in 1× PBS) for 30 minutes in a 37°C water bath prior to 40 μm strainer filtration and RBC lysis.

### Analysis of donor-reactive T cells by mixed lymphocyte reaction.

Spleen cells from transplant recipient mice were isolated in single-cell suspension and cocultured in 96-well cell culture–treated round-bottom plates in 1:1 ratio (200,000:200,000) with T cell–depleted spleen cells from donor-origin mice (or syngeneic control APCs) in complete RPMI (Gibco, Thermo Fisher Scientific) plus 10% FBS, L-glutamine, sodium pyruvate, nonessential amino acids, penicillin/streptomycin, and β-mercaptoethanol) in a 37°C incubator (5% CO_2_) overnight (18–22 hours). The following morning, GolgiPlug (Life Technologies) was added (final dilution 1:1000) and incubated for 4 hours. Positive control wells were additionally treated with PMA (10 μg/mL) and ionomycin (100 μg/mL, Sigma-Aldrich) during GolgiPlug incubation. Cells were incubated with fluorophore-conjugated antibodies and analyzed by flow cytometry. Absolute numbers of cells per spleen were calculated by multiplying the frequencies by the numbers of cells in the spleen.

### Analysis of graft-infiltrating cells.

Cell suspensions of mouse heart allografts were prepared as described above. Graft-infiltrating lymphocytes and myeloid cells were analyzed by flow cytometry using fluorophore-conjugated antibodies to cell-surface and intracellular markers. Donor reactivity of graft-infiltrating T cells was characterized by cytokine production and degranulation. Single-cell suspensions were cultured in complete RPMI in the presence of APC-conjugated CD107a (eBio1D4B) and CD107b (eBioABL-93) (eBioscience, Affymetrix) as well as GolgiPlug (final dilution 1:1000) for 4 hours (37°C, 5% CO_2_). Positive control wells were additionally treated with PMA (10 μg/mL) and ionomycin (100 μg/mL) during the 4-hour incubation. Cells were then incubated with fluorophore-conjugated antibodies and analyzed by flow cytometry.

### In vivo Treg expansion assay.

CD4^+^ T cells were isolated (>97% purity) from naive B6 TEa mice by negative selection (EasySep Mouse CD4^+^ T cell Isolation kit 19852, STEMCELL). Aliquots were stained for Foxp3 expression. Next, 20 × 10^6^ TEa^+^ CD4^+^ cells were adoptively transferred, via retro-orbital injection, into recipients on the day prior to transplantation with BALB/c hearts. Recipient mice were treated with CTLA4-Ig and/or EPO as described above. Spleen cells were harvested on postoperative day 14 and analyzed for Foxp3 expression within the CD4^+^Vα2^+^TCR^+^ gate.

### Gene expression profiling by NanoString.

B6 mice were immunized with 20 × 10^6^ BALB/c splenocytes. Recipient mice were treated with CTLA4-Ig (150 μg i.p. once on day 2, Abatacept, Bristol-Myers Squibb) with or without recombinant EPO (5000 U/kg i.p. daily, epoetin alfa, Amgen). On day 7, we isolated splenic CD11b^+^ myeloid cells by positive selection (kit 18970, STEMCELL). RNA was isolated using TRIzol (Thermo Fisher Scientific) and cleaned using RNeasy Mini Kit (Qiagen). Gene expression profiling was then performed using the nCounter Myeloid Innate Immunity panel (NanoString Technologies). Raw counts were imported into the R package Limma for normalization and determination of differential expression. The gene expression data have been deposited in the NCBI Gene Expression Omnibus (GEO GSE199270).

### Signaling assay for EPOR conditional knockout validation.

Single-cell suspensions of total splenocytes were plated in 96-well round-bottom plates (1 × 10^6^ cells per well in 200 μL complete RPMI) and stimulated with either anti-CD3 antibody clone 145-2C11 (1 μg/mL final) (Invitrogen) for 18 hours in a 37°C (5% CO_2_) incubator. Cells were then washed and resuspended in 200 μL HL-1 medium (Lonza) (supplemented with L-glutamine and penicillin/streptomycin) and rested in a 37°C (5% CO_2_) incubator for 2 hours prior to stimulation. The cells were stimulated with recombinant EPO (1000 U/mL) (epoetin alfa, Amgen) for 30 minutes. Phospho-STAT5 was quantified by phospho-flow cytometry.

### Antibodies and reagents.

Flow cytometry antibodies against CD3 (clone 17A2), CD4 (clone GK1.5), CD40 (clone 3/23), CD45.1 (clone A20), and CD45.2 (clone 104) were purchased from BioLegend; CD8 (clone 2.43) and CD62L (clone MEL-14) were purchased from Tonbo Biosciences; CD11b (clone M1/70), CD44 (clone IM7), CD45 (clone 30-F11), FoxP3 (clone FJK-16s), IFN-γ (clone XMG1.2), and eFluor 780 fixable viability dye were purchased from Invitrogen; CD11c (clone N418), H-2K^d^ (clone SF1-1.1.1), IL-4 (clone 11B11), IL-17a (clone eBio17B7), T-bet (clone eBio4B10), and TNF-α (clone MP6-XT22) were purchased from eBioscience (Affymetrix); CD80 (clone 16-10A1), CD86 (clone GL1), and BD Phosflow Alexa Fluor 647 Mouse Anti-Stat5 (pY694) were purchased from BD Biosciences. Fc receptors were blocked with purified anti-CD16/anti-CD32 (Fc Shield; 2.4G2; Tonbo Biosciences). All antibodies or dyes were used at a dilution of 1:400, except eFluor 780 fixable viability dye (1:1000). Intracellular cytokine and transcription factor detection were performed using FoxP3 staining buffer set per the manufacturer’s instructions (Affymetrix). Phospho-protein detection was performed using 1% paraformaldehyde fixation buffer (Electron Microscopy Sciences), BD Phosflow Perm buffer III (BD Biosciences), and wash/staining buffer consisting of 1% w/v BSA (Sigma-Aldrich) in 1× PBS (Life Technologies). All incubations were performed in the dark on ice for 20 minutes except p-STAT5 (1 hour at room temperature).

### Flow cytometric analysis.

Flow cytometric analysis was performed on a FACSCanto II (BD Biosciences) and analyzed using Cytobank or FlowJo software (Becton Dickinson).

### Statistics.

Statistical analysis was performed with GraphPad Prism software version 5. Differences between graft survival rates were assessed by Mantel-Cox log-rank test. For comparisons of 2 groups, we used Student’s *t* test (unpaired, 2-tailed). For experiments involving 3 or more groups, we used 1-way ANOVA corrected for multiple comparisons. All experiments were repeated at least twice. Data are presented as mean values with SD. *P* values of less than 0.05 were considered significant. To obtain differentially expressed genes from NanoString analysis, we performed a Limma test (54) between groups. Differentially expressed genes were identified at *P* value less than 0.05, and then were subjected to Gene Ontology function analysis (55).

### Study approval.

The murine studies were performed at the Icahn School of Medicine under IACUC-approved protocol number 0082, originally approved in May 2015, renewed for May 11, 2019, through May 11, 2022.

## Author contributions

PSH and PC (co–senior authors) jointly conceived and provided oversight for the project. They reviewed all data and contributed to data analysis, writing, editing, and figure production. Order of senior authorship was agreed upon by mutual discussion. JCM, PC, and PSH cofunded the project. JKH and SB are listed as co–first authors. The order of their authorship was decided upon via mutual discussion: JKH initiated the project, performed and analyzed the majority of experiments, analyzed data, and prepared figures (Figures 1–4). SB took over the work when JKH left, completed all ongoing experiments and analyses, repeated several experiments to increase numbers, assisted with the gene expression analyses, and edited the manuscript. PSH developed the conditional EPOR-knockout mice. JKH and SB both contributed to writing and editing the manuscript. KSK performed the NanoString analyses in Figure 5. RLF oversaw the NanoString experiments and contributed to writing and editing of the manuscript. ZY and WZ performed the gene expression analyses and statistical comparisons for the results shown in Figure 5. VIP and YL performed the heterotopic heart transplants in mice.

## Supplementary Material

Supplemental data

## Version 1 04/07/2022

In-Press Preview

## Version 2 05/23/2022

Electronic publication
