# Supplementary material for: Linking erythropoietin to Treg-dependent allograft survival through myeloid cells
Source: JCI Insight. 2022 May 23;7(10):e158856. doi: 10.1172/jci.insight.158856 (PMC9220923; doi:10.1172/jci.insight.158856)
Supplement: Supplemental data [file jciinsight-7-158856-s137.pdf]

Supplemental Figure 1

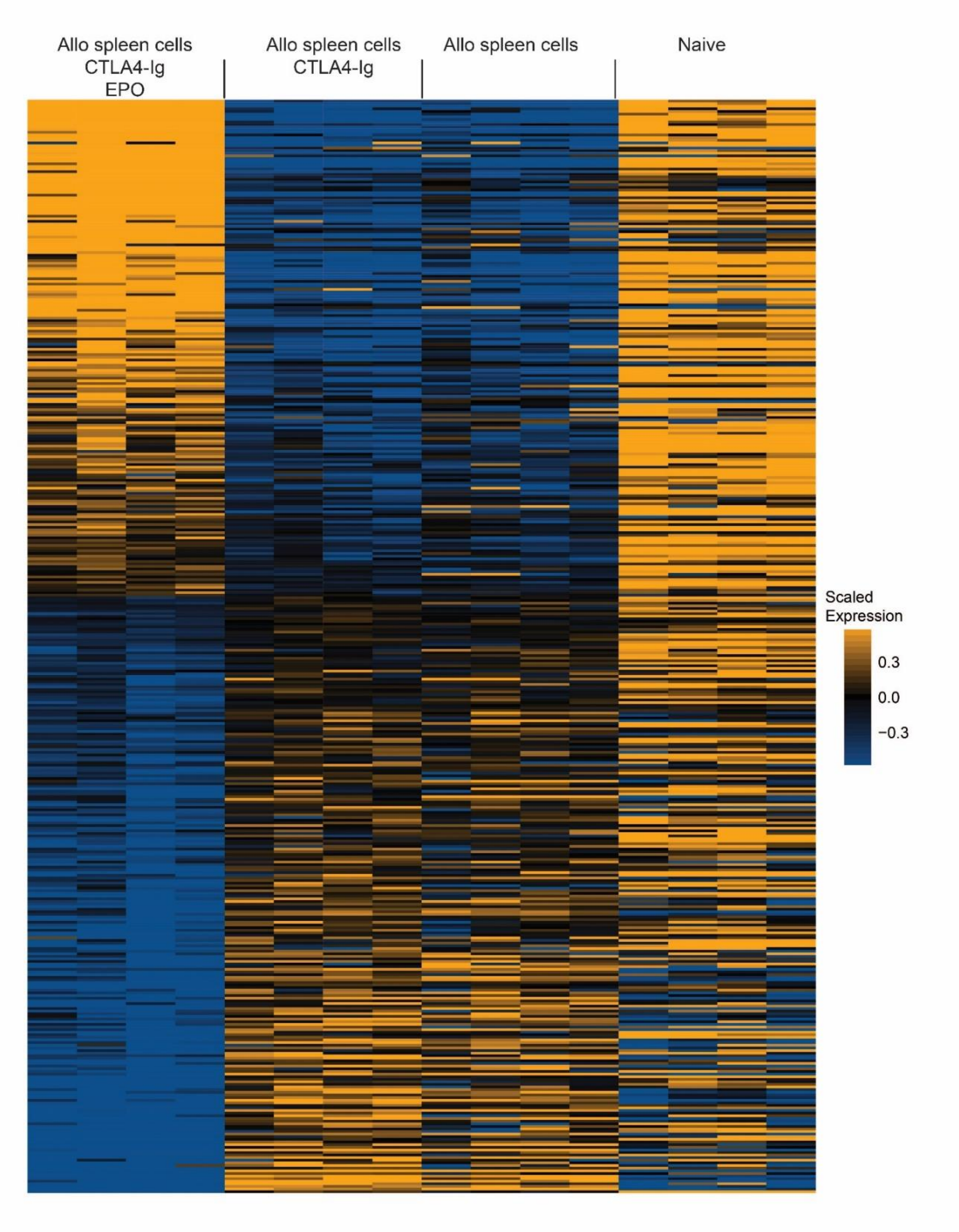

Muti-group heatmap demonstration of all differentially expressed genes from Limma test between splenic macrophages isolated from animals treated with donor spleen cells, CTLA4-Ig and EPO vs donor spleen cells + CTLA4-Ig alone (see Schematic in Figure 5A).
